# Exploring the methylation status of CFTR and PKIA genes as potential biomarkers for lung adenocarcinoma

**DOI:** 10.1186/s13023-023-02807-1

**Published:** 2023-08-29

**Authors:** Bowen Xu, Jingang Zhang, Weigang Chen, Wei Cai

**Affiliations:** 1 Xuzhou Central Hospital, Xuzhou, Jiangsu 221000 China; 2 The 2nd Medical College of Binzhou Medical University, Yantai, Shandong 264000 China; 3 Weihai Second Hospital affiliated to Qingdao University, Weihai, Shandong 264200 China

**Keywords:** DNA methylation, Lung adenocarcinoma, Prognostic signature, TCGA, GEO

## Abstract

**Background:**

One of the most prevalent cancers in the world is lung cancer, with adenocarcinoma (LUAD) making up a significant portion of cases. According to the National Cancer Institute (NCI), there are new cases and fatality rates per 100,000 individuals as follows: New instances of lung and bronchial cancer occur annually at a rate of 50.0 per 100,000 persons. The yearly death rate for men and women is 35.0 per 100,000. DNA methylation is one of the earliest discovered and widely studied epigenetic regulatory mechanisms, and its abnormality is closely related to the occurrence and development of cancer. However, the prognostic value of DNA methylation and LUAD needs to be further explored to improve the survival prediction of LUAD patients.

**Methods:**

The transcriptome data and clinical data of LUAD were downloaded from TCGA and GEO databases, and the Illumina Human Methylation450 array (450k array) data were downloaded from the TCGA database. Firstly, the intersection of the expressed genes of the two databases is corrected, the differential analysis is performed, and the methylation data is evaluated by the MethylMix package to obtain differentially methylated genes. Independent prognostic genes were screened out using univariate and multivariate Cox regression analysis, and a methylation prognostic model was developed using univariate Cox analysis and validated with the GSE30219 dataset in the GEO database. Survival analysis between methylation high-risk and low-risk groups was performed and a methylation-based gene prognostic model was constructed. Finally, the prediction of potential drugs associated with the LUAD gene signature using Drug Sensitivity Genomics in Cancer (GDSC).

**Results:**

In this study, a total of 555 samples from the TCGA database and 307 samples from GSE30219 were included, and a total of 24 differential methylation driver genes were identified. Univariate and multivariate Cox regression analyzes were used to screen out independent prognostic genes, involving 2 genes: CFTR, PKIA. Survival analysis was different between the methylation high-risk group and the low-risk group, the CFTR high methylation group and the low methylation group were poor, and the opposite was true for PKIA.

**Conclusions:**

Our study revealed that the methylation status of CFTR and PKIA can serve as potential prognostic biomarkers and therapeutic targets in lung cancer.

**Supplementary Information:**

The online version contains supplementary material available at 10.1186/s13023-023-02807-1.

## Introduction

The World Health Organization (WHO) notes that lung cancer is one of the most prevalent malignancies in developing nations, where its prevalence is also on the rise as a result of things like air pollution and smoking [1]. The National Cancer Institute (NCI) reports new cases and mortality rates per 100,000 people: The annual incidence of new lung and bronchial cancers is 50.0 cases per 100,000 people. The annual male and female mortality rate is 35.0 per 100,000,Based on 2017–2019 data, approximately 6.1% of men and women will be diagnosed with lung and bronchial cancer at some point in their lifetime [2]. Lung cancer is mainly divided into non-small cell lung cancer (NSCLC) and small cell lung cancer (SCLC). NSCLC accounts for approximately 85% of lung cancers, with lung adenocarcinoma (LUAD) and lung squamous cell carcinoma (LUSC) being the most common subtypes [3]. People who die of lung cancer are often diagnosed at an advanced stage. Therefore, an early lung cancer diagnosis is essential, requiring sensitive and specific biomarkers for early diagnosis.

DNA methylation is one of the earliest discovered and widely studied epigenetic regulatory mechanisms [4]. DNA methylation refers to the formation of 5- methylcytosine (5mC) by transferring the methyl group to the 5-carbon position of the cytosine ring with s- adenosylmethionine (SAM) as the methyl donor [5]. Abnormal DNA methylation is closely related to the occurrence and development of cancer [6], and changes in DNA methylation have been observed in various types of cancer [7]. Hypomethylation of oncogenes and hypermethylation of tumor suppressor genes are pathogenic mechanisms in most tumors [8]. The former is through genome-wide hypomethylation, which plays a stabilizing role in the structure of heterochromatin, and the latter is through the hypermethylation of promoter genes to inhibit the expression of oncogenes [9]. Aberrant DNA methylation contributes to the development and progression of lung cancer [10]. Abnormal DNA methylation patterns were observed in lung cancer cells compared with normal lung tissue [11].

In this study, we obtained the transcriptional data and clinical data of LUAD patients from TCGA and GEO, and conducted a comprehensive analysis, and finally determined a set of DNA methylation features of the CFTR and PKIA genes. We performed a receiver operating characteristic curve to verify the ability of the identified DNA methylation profiles to predict the survival of LUAD. In addition, we carried out immunohistochemical chemistry, and the results showed that these genes were different from normal people and tumor patients.

## Materials and methods

### Data collection

DNA methylation data was downloaded from TCGA database((https://portal.gdc.cancer.gov/)).DNA methylation profile was measured experimentally using the Illumina Infinium HumanMethylation 450 platform. The level of DNA methylation is express as a beta value. The transcriptome profiling data and clinical information also were downloaded from TCGA and GEO database (https://www.ncbi.nlm.nih.gov/geo/). The DNA methylation data contains 466 samples which include 29 normal samples 437 tumor samples and from TCGA. Gene expression data were downloaded from TCGA and GEO (GSE30219). TCGA included 54 normal and 501 tumor samples, and GEO included 14 normal and 293 tumor samples. Data analysis and collation were carried out using R software(version 4.2.1)and perl software(v5.30.0).

### Screening of differentially expressed genes(DEGs)

The transcriptome analysis data and clinical information from TCGA and GEO were transformed into a gene expression matrix using Perl software, and R was used to intersect the two expression matrices of TCGA and GEO to obtain the intersection gene expression level. Batch corrections were performed on both datasets simultaneously to obtain normalized expression levels. Using the “Limma” and “Pheatmap” package in R software, log2 conversion and DEGs screening of TCGA normalize data were performed. |log2FC|>0.585 and adjust P-value < 0.05 were regarded as the statistical significance threshold level of DEGs samples.

### Differential methylation analysis

Use the “Limma” package of R software to read differential gene expression files and methylation data files, extract the expression and methylation data of normal samples and tumor samples, and intersect the two to obtain four files: Expression files and methylation files for normal and tumor samples. Methylation-driven genes need to meet the following conditions: (1) The gene expression level is different between the normal group and the tumor group. (2) The gene methylation level is different and negatively correlated between the normal group and the tumor group. Use the “MethylMix” package of R software to screen methylation-driven genes, Cox regression analysis was used to study the correlation between gene methylation and expression, and 24 genes were obtained.

### Construction of prognostic risk model

To correlate the expression of DNA methylation regulators with overall survival (OS), we performed a univariate Cox regression analysis. In the LASSO Cox regression algorithm, the optimal penalty parameter λ and the corresponding coefficient criterion are determined according to the minimum criterion through cross-validation. Patients were divided into the low methylation risk group and a high-risk group according to the average risk score [12]. We used survival curves to compare overall survival (OS) between the two groups. Risk curves were plotted to show the relationship between risk assessment, survival status, and the two gene methylation levels. To investigate whether risk score could be used as an independent factor, we performed univariate and multivariate Cox regression analysis using the “survival” R-package.

### Establishment and evaluation of Nomogram for survival rate prediction

To accurately predict OS at 1, 3, and 5 years, clinical information with independent predictors was combined with risk scores to create prognostic nomograms. Harrell’s Concordance Index (C-Index) was used to assess forecast accuracy. The c-index ranges from 0.5 (no predictive power) to 1 (perfect prediction). Assess the performance of the nomogram using the calibration plot. Each patient was assigned an overall nomogram score, the nomoscore, and the quantiles of the nomo score were used as cut-off points to classify patients into three risk groups. KM curves further check the performance of the nomogram.

### Gene set enrichment analysis

Functional enrichment of gene expression data was interpreted using gene set enrichment analysis (GSEA). The method analyzes the genome to determine whether they are statistically significantly different under the two biological conditions. Within the “Molecular Signatures Database”(http://www.gsea-msigdb.org/gsea/msigdb/index.jsp) of c2.cp.kegg. symbols and c5.go.symbols by GSEA with R software, underlying mechanisms were studied. The random sample permutation number was set as 500, and the significance threshold p < 0.050.

### Drug sensitivity prediction

The GDSC(https://www.cancerrxgene.org/) database, the largest public resource of molecular indicators of cancer drug sensitivity and treatment response, for differential analysis of drug sensitivity [13]. The drug susceptibility values (IC50) of 198 drugs were downloaded from the GDSC database. Drug response prediction was evaluated using the ‘oncoPredict’, ‘limma’ and ‘paralle’ packages in R, and the accuracy of the prediction was estimated by cross-validation.

### Immunohistochemistry

Sections were deparaffinized, followed by antigen retrieval in citrate buffer and blocking with 3% hydrogen peroxide. Subsequently, block sections with 3% fetal calf serum for 60 min, and then use antibodies (rabbit polyclonal anti-CFTR antibody, 1:100; Fine Test, FNab01623, China. Rabbit polyclonal anti-PKIA antibody 1:100; CUSABIO, CSB-PA030217, China) or fetal bovine serum (negative control) were incubated overnight at 4 °C. The next day, the secondary antibody [1:400, HRP-conjugated Goat Anti-Rabbit IgG, Sangon Biotech, NO. D110058), China] was incubated for 30 min at 37 °C. After washing, immunoreactivity was visualized by incubating tissue sections with a DAB staining kit (GTVision^TM^+Detection System/Mo&Rb, GK600710) and counterstaining with hematoxylin. Immunoreactive cells are stained tan.

## Results

### Identification of DEGs in LUAD

Transcriptome profiling was obtained from TCGA for lung cancer tissues (n = 501) and non-tumor tissues (n = 54) (supplement 1), GEO database expression files (GSE30219) included 14 normal samples and 293 tumor samples (supplement 2, 3). The intersection of genes is taken, and the expression levels of the intersection genes in the two databases are obtained respectively, and batch correction is performed. TCGA-corrected expression files were analyzed using FDR < 0.05 and |log2 FC| > 0.585 as thresholds. A total of 4471 genes were screened, of which 1900 genes were up-regulated and 2571 genes were down-regulated for subsequent analysis (supplement 4). The identified DEGs were visualized using a heatmap and a volcano map(supplement Fig. 1sA, B), where the heatmap shows the most significantly upregulated and downregulated 50 genes.

### Identification of DNA methylation-driven genes in LUAD

**Fig. 1:**
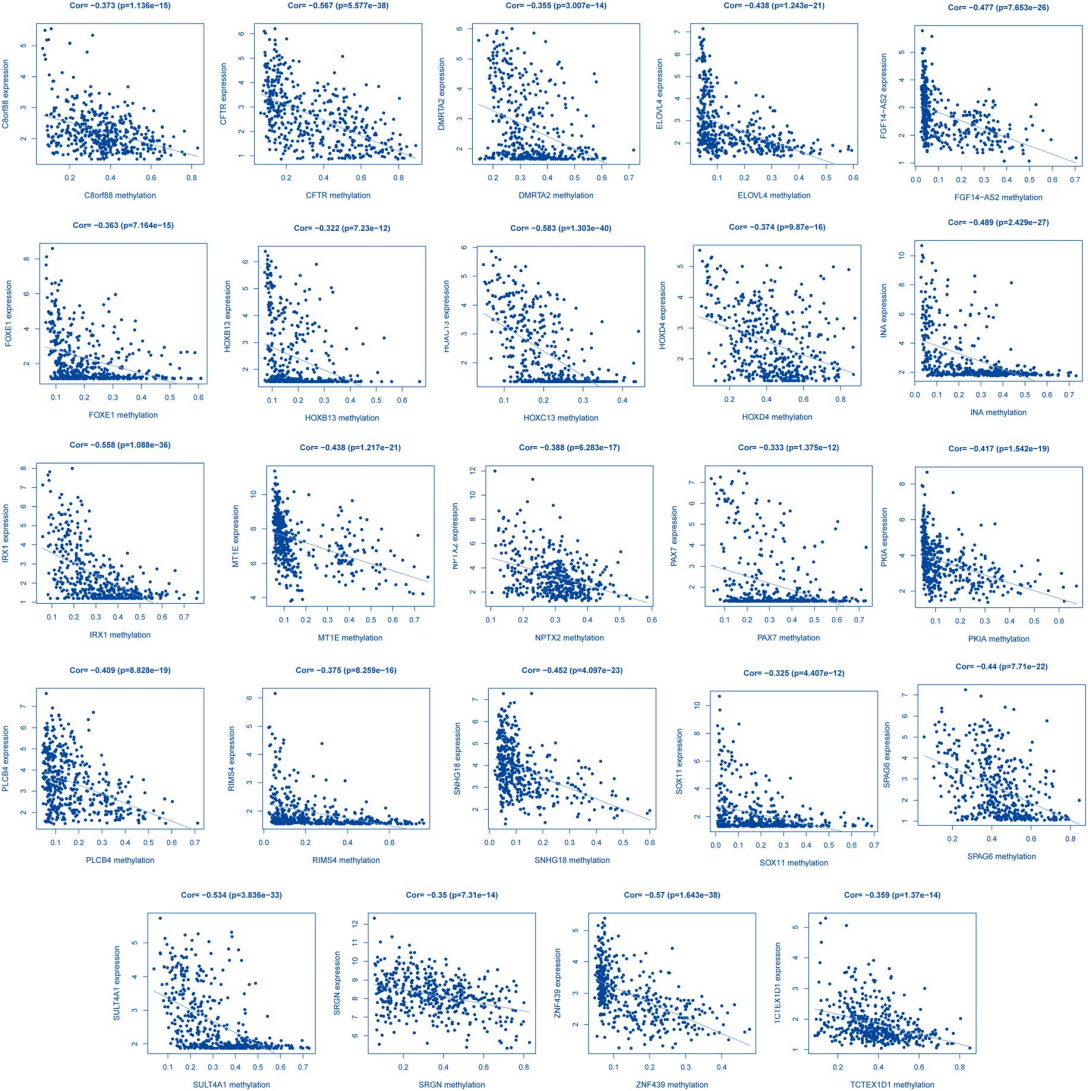
Correlation between methylation and expression in LUAD. The x-axis is an expression, the y‐axis is methylation

To identify DNA methylation driver genes in LUAD, gene expression and DNA methylation data of 2571 DEGs from 466 clinical samples from TCGA (437 LUAD samples and 29 non-tumor samples) (supplement 5) were included in the methylation analysis. The inclusion criteria are: (1) There is a difference in gene expression between the normal group and the tumor group; (2) The gene methylation level is different in the normal group; (3) There is a negative correlation between the gene methylation level and gene expression, 24 methylation-driven genes were screened out(Figs. 1 and supplement Fig. ">2s)( Table 1). Then, we plotted the expression data heatmap and methylation heatmap of these 24 genes(supplement Fig. 3sA, B).

**Table 1:** 24 methylation-driven gene expression levels

Gene	NormalMean	TumorMean	LogFC	pValue	cor	corPavlue
DMRTA2	0.15586033	0.33573431	1.107066201	4.15E-19	-0.35502	3.01E-14
TCTEX1D1	0.110839429	0.388068085	1.807838602	3.39E-18	-0.35943	1.37E-14
PAX7	0.101592835	0.322152992	1.664947334	2.14E-16	-0.33255	1.37E-12
NPTX2	0.149705162	0.299575624	1.000796276	4.27E-16	-0.38793	6.28E-17
SPAG6	0.189199309	0.429502219	1.182758766	3.65E-15	-0.44006	7.71E-22
INA	0.048689548	0.261957419	2.427648315	3.07E-14	-0.48941	2.43E-27
IRX1	0.129256328	0.311996697	1.271295843	7.25E-14	-0.55832	1.09E-36
HOXD4	0.159162933	0.421297423	1.404334701	1.24E-13	-0.3737	9.87E-16
HOXC13	0.103351602	0.207111123	1.002844276	2.63E-13	-0.58306	1.30E-40
SOX11	0.027224838	0.189542612	2.799526849	7.14E-13	-0.32532	4.41E-12
C8orf88	0.153447229	0.352259416	1.198895678	1.41E-12	-0.37296	1.14E-15
SULT4A1	0.145358808	0.315548904	1.118245117	6.23E-12	-0.534	3.84E-33
HOXB13	0.096330073	0.195210377	1.018971577	1.59E-11	-0.32219	7.23E-12
RIMS4	0.066327817	0.252814782	1.930394873	2.21E-11	-0.37464	8.26E-16
CFTR	0.121184164	0.353033518	1.542603981	2.37E-11	-0.5667	5.58E-38
PLCB4	0.068438447	0.17990335	1.394343117	5.88E-11	-0.40867	8.83E-19
FOXE1	0.106971686	0.216507905	1.017190717	8.89E-10	-0.36302	7.16E-15
SNHG18	0.048018969	0.123543121	1.363338348	1.19E-08	-0.45223	4.10E-23
SRGN	0.148781488	0.344444865	1.211078035	2.21E-08	-0.34996	7.31E-14
PKIA	0.059493661	0.13919659	1.226316018	1.13E-07	-0.41676	1.54E-19
ZNF439	0.067991441	0.145539526	1.097985967	1.14E-07	-0.57007	1.64E-38
FGF14-AS2	0.036592022	0.126262565	1.786825914	2.76E-07	-0.47675	7.65E-26
MT1E	0.071121203	0.167446392	1.235347652	3.22E-07	-0.43812	1.22E-21
ELOVL4	0.067494181	0.153384926	1.18432169	0.0008397	-0.43803	1.24E-21

**Fig. 2:**
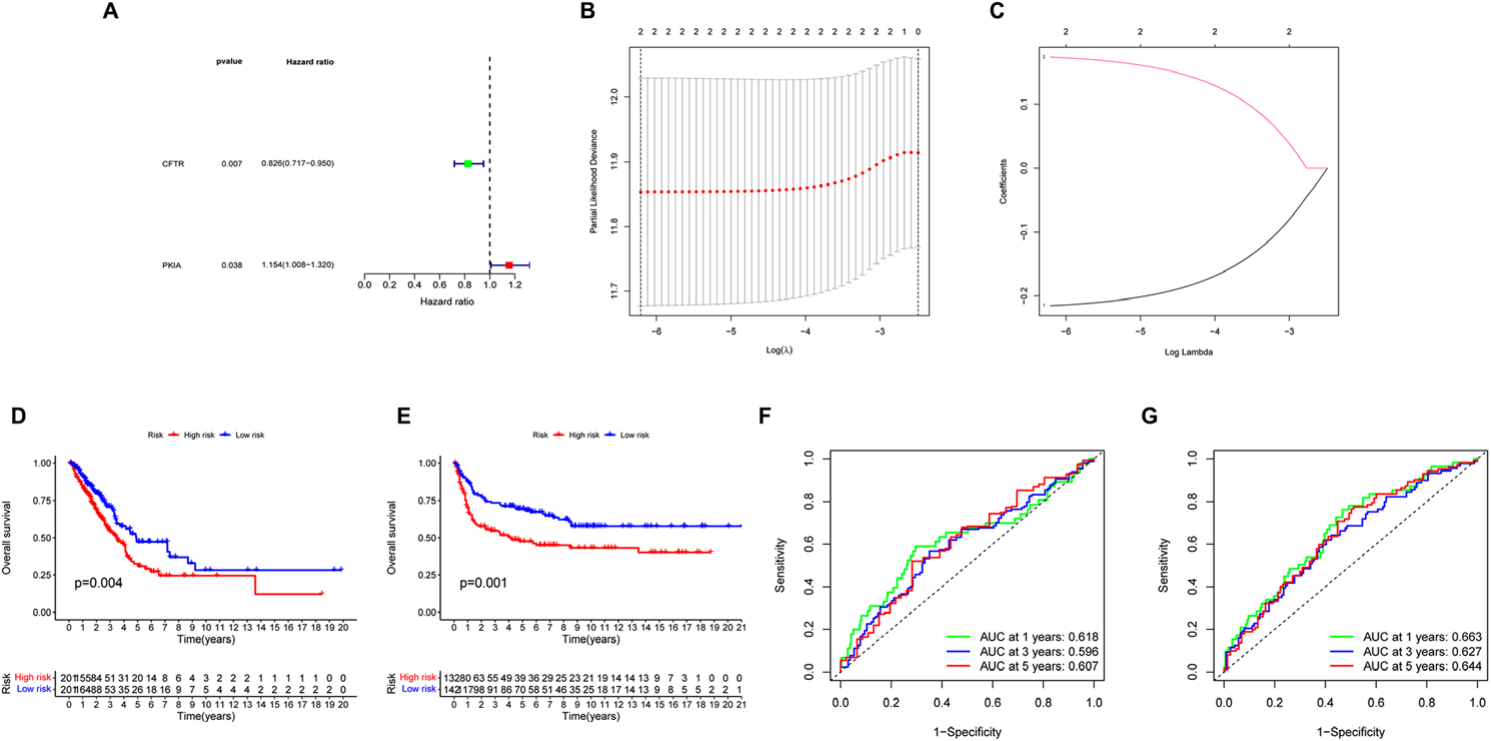
Construction of prognostic risk profiles for patients with two survival-related genes LUAD. **(A)** Forest map of screening prognostic genes. **(B,C)** Using LASSO Cox regression, two m5C RNA methylation regulators were selected for risk coefficient calculation. **(D,E)** Survival analysis of TCGA and GEO in high-risk and low-risk population. **(F,G)** Time -ROC curve analysis of 1-,3-,and 5-year risk characteristics of TCGA and GEO.

### Construction and validation of prognostic models of DNA methylation-driven genes

We analyzed the prognosis of 24 genes and observed the effect of their expression on the prognosis of LUAD patients. Hazard ratio(HR) > 1 represents high-risk genes, HR < 1 represents low-risk genes, and found that CFTR and PKIA genes (P < 0.05) have prognostic significance in lung cancer(Fig. 2A). Using the methylation regulators of CFTR and PKIA, we performed a LASSO Cox regression algorithm and constructed a prognostic model based on these two regulators (Fig. 2B,C). Finally, we identified the factors that will be used to calculate the risk score after cross-validation. The coefficients of CFTR and PKIA are − 0.22127 and 0.17919, respectively (Table 2).The risk score for each patient with TNBC was calculated using the following formula: risk score = − 0.22127 × CFTR + 0.17919 × PKIA. According to the median risk score, all patients with LUAD were divided into a low-risk group and a high-risk group. In both the TCGA and GEO databases, the OS of patients in the high-risk group was lower than that in the low-risk group (Fig. 2D, E). To evaluate the specificity and sensitivity of risk characteristics in predicting the prognosis of LUAD, we performed a time-dependent receiver operating characteristic curve (ROC) analysis in the TCGA database and the GEO data set, and the areas under the ROC curve (AUC) of the 1, 3, and 5-year analyses were 0.618,0.596,0.607,0.663,0.627,0.644,respectively (Fig. 2F,G),suggesting that our risk characteristics can be used to predict the prognosis of LUAD.

**Table 2:** Risk scores for CFTR and PKIA

ID	coef
CFTR	-0.221270655088416
PKIA	0.179195151042991

### Relationship between clinical features and prognostic risk score

**Fig. 3:**
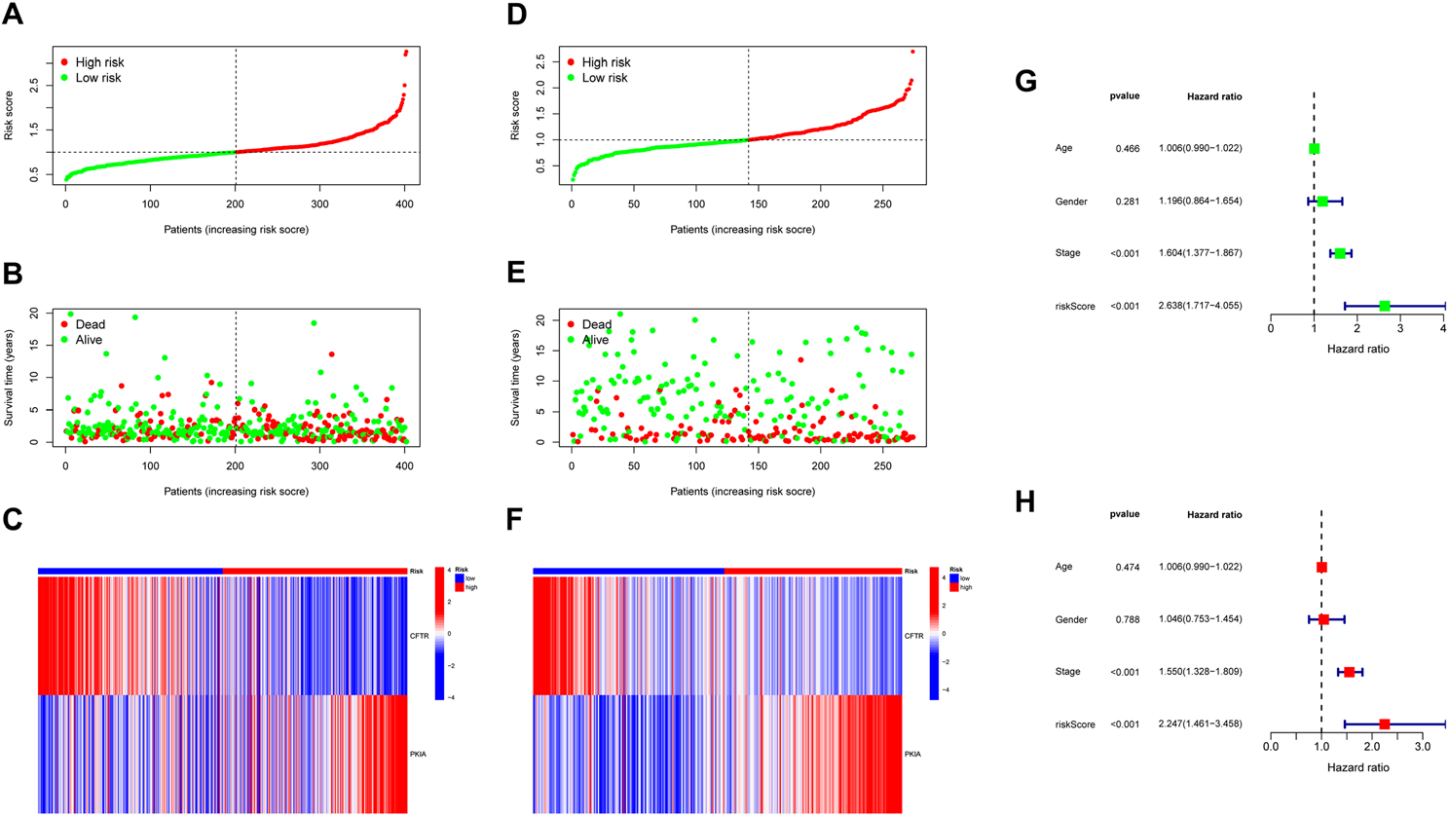
**(A,D)** Risk score distribution of TCGA and GEO. **(B,E)** Survival status scatter plots of TCGA and GEO. **(C,F)**Heat maps show differences in CFTR and PKIA expression between the high-and low-risk groups of TCGA and GEO. **(G)** The p-value, HR value, and 95% confidence interval of the DNA methylation regulators analyzed using univariate Cox regression analysis **(H)** Multivariate Cox regression analysis

TCGA and GEO risk score plots, survival time and status plots are shown in Fig. 3A, B, D, and E. In addition, these 2 independent risk genes were displayed in a heat map to show the difference in expression levels between high-risk and low-risk groups (Fig. 3C,F). Then we performed univariate and multivariate Cox regression analysis, riskscore was significantly correlated with OS(p < 0.001). At the same time, univariate and multivariate Cox regression analysis also showed that histological stage was also associated with OS (p < 0.001) (Fig. 3G,H). These results suggest that risk models based on these two methylation regulators can serve as independent prognostic factors for LUAC patients.

### Establishment of a prognostic nomogram for prediction of LUAD OS

**Fig. 4:**
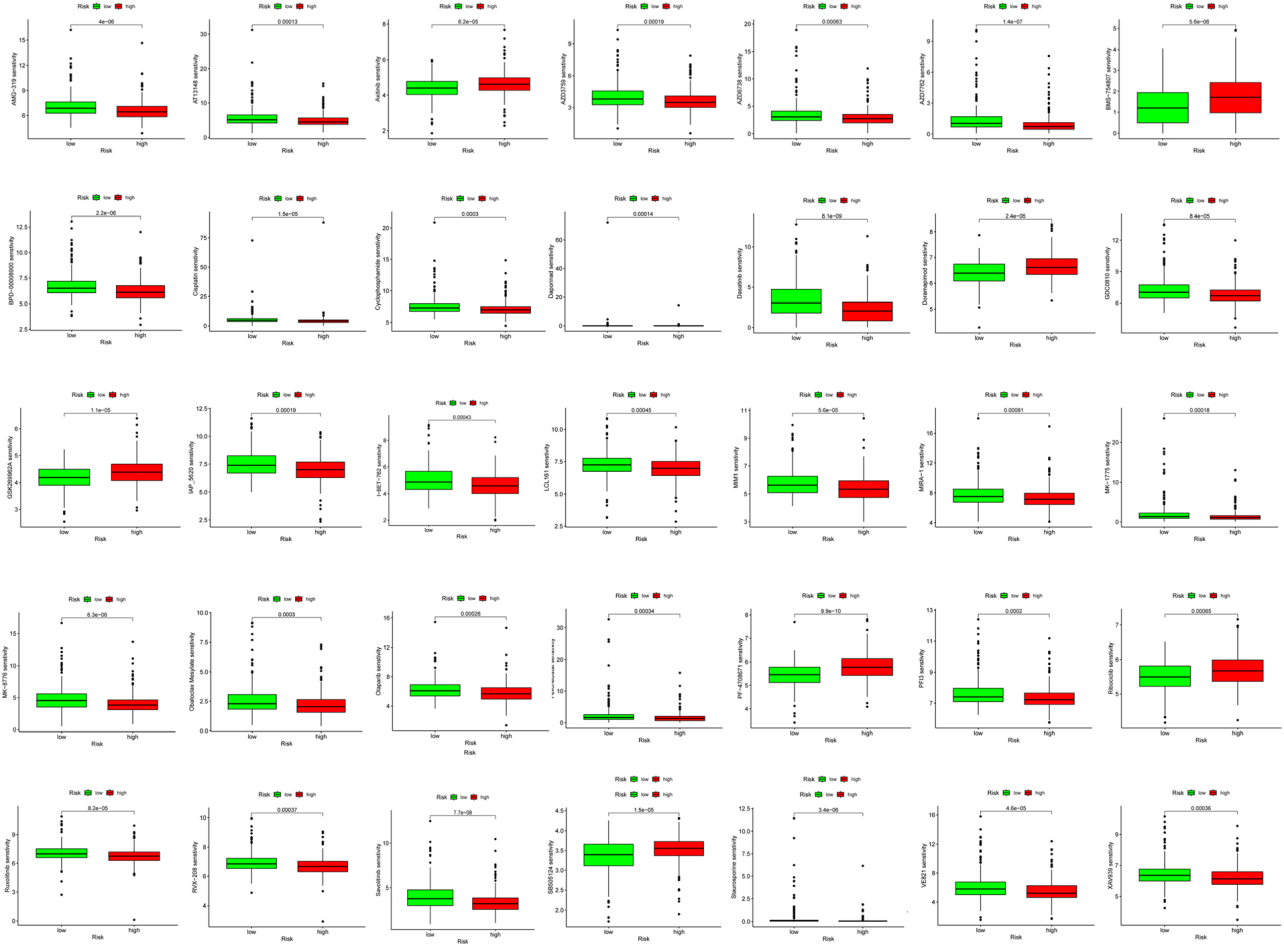
**(A)**Survival analysis of CFTR expression levels. **(B)** Survival analysis of PKIA expression levels. **(C)**Survival analysis of CFTR methylation levels. **(D)**Survival analysis of PKIA methylation levels. **(E)**A correction curve for assessing the consistency between the prediction of the prognostic model and the actual survival rate. **(F)**Survival probability nomogram of all independent predictive variables based on TCGA data set

Survival analyses of model gene expression and methylation levels showed that CFTR and PKIA were associated with survival in patients with LUAD (Fig. 4A-D).In order to manage the clinical prognosis of lung cancer patients and provide clinicians with a quantitative method to predict the probability of one-,three-and five-year survival time of individuals, we established a prognostic nomogram that integrates clinical pathology independent risk factors with a prognostic model (Fig. 4F). Based on the above predictive model, the calibration curve of the nomogram shows that the predicted OS rates at 1, 3, and 5 years are in good consistency with the final results (Fig. 4E).

### Biological mechanisms of model gene function

To investigate potential CFTR- and PKIA-related functions and signaling pathways, we performed GSEA. After GO and KEGG analysis, GSEA (supplement Fig. 4sA-D) found that new model genes were overexpressed in high-risk and low-risk groups, and regulated multiple biological processes, such as antigen processing and presentation, cell cycle, DNA replication, aldosterone regulates sodium reabsorption, juvenile diabetes, and tyrosine metabolism.

### Chemotherapy strategy of LUAD high and low-risk group

**Fig. 5:**
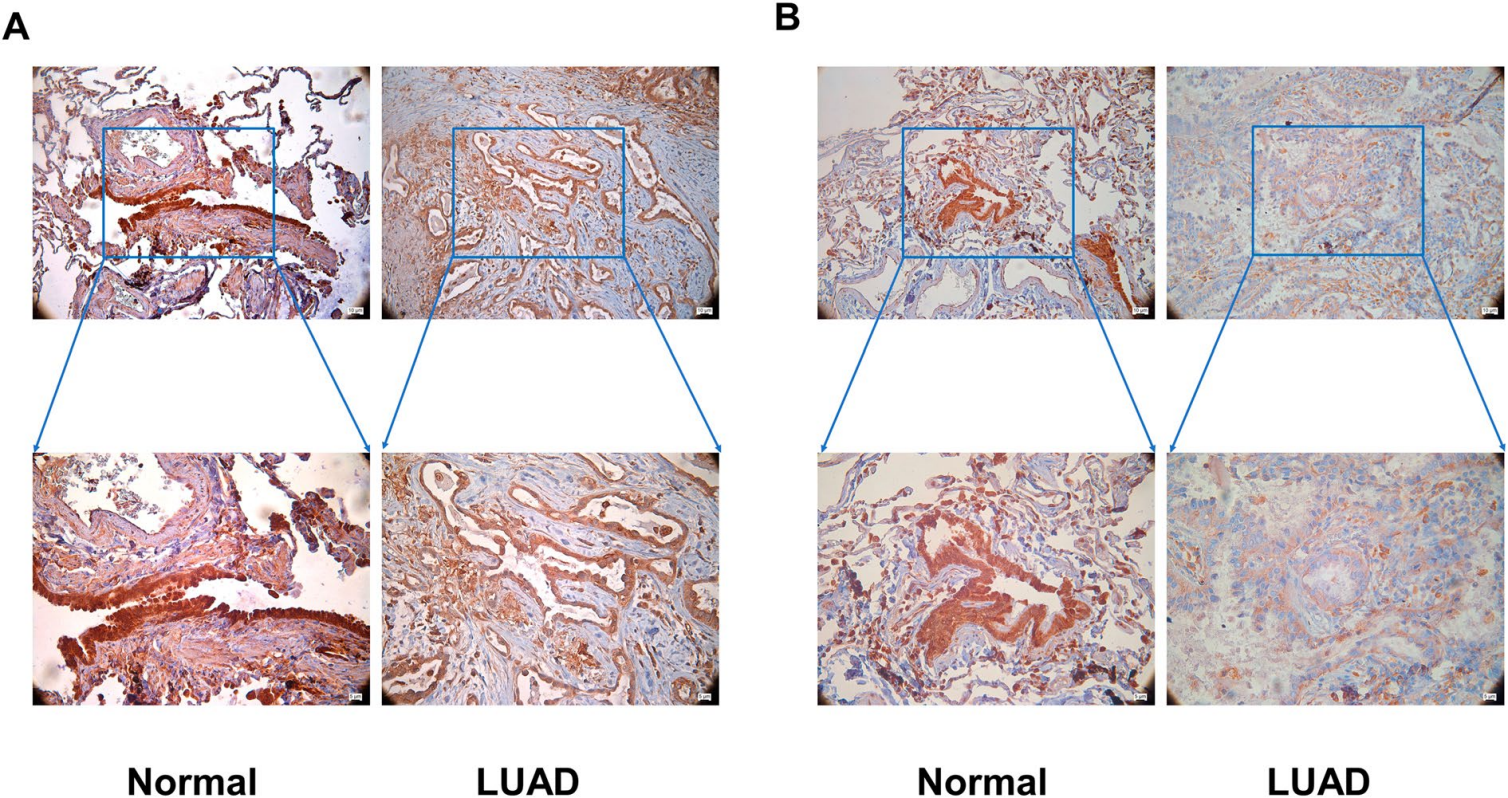
Boxplot of susceptibility to commonly used chemodrugs evaluated between high-risk and low-risk groups by analysis of cell line data from the GDSC database

The Oncopredict software package was used to predict the drug sensitivity scores of the two groups with high and low LUAD scores. Based on the GSDC database, the correlation between the expressions of CFTR and PKIA and the sensitivity to antitumor drugs was analyzed. The results showed that the lower the drug susceptibility score, the more sensitive to drug treatment. The results show that our signature genes are associated with the response to 35 anti-tumor drugs and have potential anti-tumor value (Fig. 5).

### Expression of CFTR and PKIA in LUAD tissues

**Fig. 6:**
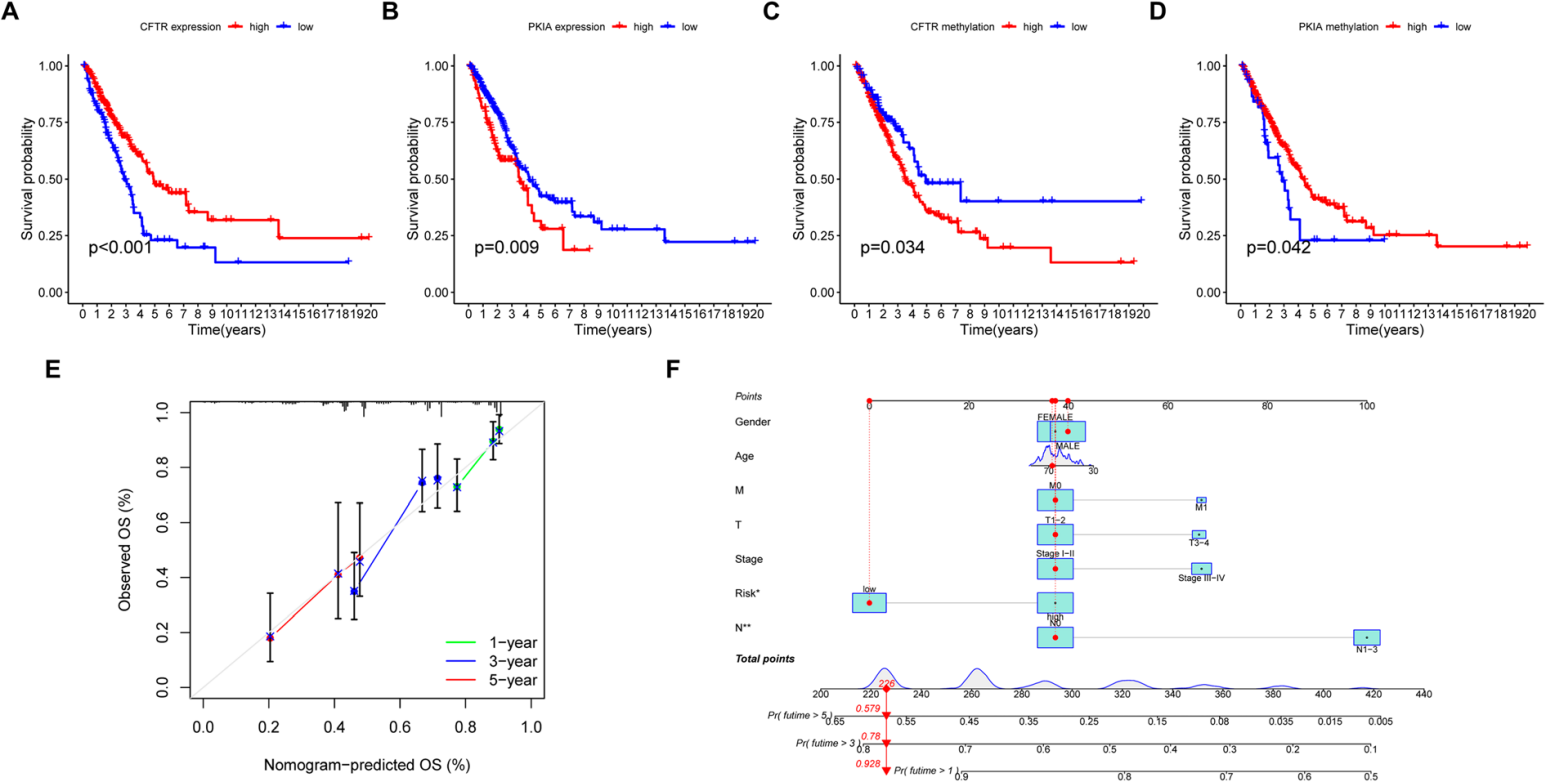
Immunohistochemistry: **(A)** CFTR is strongly expressed in adjacent normal lung tissue, and decreased expressed in LUAD (magnification ×20, magnification ×40) **(B)** PKIA is strongly expressed in adjacent normal lung tissue, and in decreased expressed in LUAD (magnification × 20, magnification × 40)

The expressions of CFTR and PKIA were detected by immunohistochemical (IHC) staining using lung tissue and LUAD tissue from our hospital. The results showed that CFTR and PKIA were strongly expressed in normal lung tissue and weakly expressed in LUAD tissue(Fig. 6). These results were consistent with the results of the dataset, indicating that CFTR and PKIA played an important role in the progression of lung cancer.

## Discussion

Lung cancer is the main cause of cancer-related mortality worldwide [14]. Despite the progress in diagnosis and treatment, the five-year survival rate of lung cancer is still very low. With the advent of new sequencing technologies, genome-wide DNA methylation analysis has become possible. In the study of cancer, it is found that apparent genetic changes also play an important role in the process of tumors [15]. Aberrant DNA methylation usually occurs in the early stages of cancer, making DNA methylation biomarkers a good marker for early cancer detection [16]. DNA methylation is also associated with lung cancer prognosis and is essential for its growth and metastasis [17].In this article, we discuss the role of CFTR and PKIA methylation in lung cancer, as revealed by data analysis from TCGA and GEO. We found that the methylation of CFTR and PKIA genes correlated with the prognosis of lung cancer. They are not only differentially methylated and expressed in LUAD tumor tissues, but also correlated with the prognosis of patients. Survival curves showed that there were significant differences in survival curves between the high-risk group and the low-risk group.

CFTR is a gene that encodes a chloride channel that is critical for regulating the transport of ions and fluids across epithelial tissues [18].CFTR protein deficiency leads to excessive inflammation in the lungs, pancreas and intestines [19, 20]. Mutations in the CFTR gene are closely related to cystic fibrosis, and different types of CFTR mutations can lead to CFTR protein deficiency and functional impairment, and the severity of lung diseases varies significantly among different individuals [21]. Hypermethylation of the CFTR promoter has also been observed in head and neck cancer and non-small cell lung cancer, bladder cancer, liver cancer and breast cancer [22]. Previous articles found that hypermethylation of the CFTR promoter region can lead to a decrease in the expression of CFTR in lung cancer cells, and hypermethylation of the CFTR gene in NSCLC tissue samples was associated with a significantly reduced survival rate [23, 24], which is consistent with our conclusions.

PKIA, also known as cAMP-dependent protein kinase inhibitor α, has the function of interacting with cAMP-dependent protein kinase and inhibiting its activity [25]. PKIA is elevated in prostate cancer and associated with reduced progression-free survival, and its depletion leads to reduced tumor growth and migration, and increased susceptibility to anoikis [26]. Satarupa et al. pointed out that PIKA is an important biomarker for cervical cancer staging [27]. Another study showed that the PIKA-mRNA signature acts as a prognostic signature in thyroid cancer and is also associated with the infiltration of immune cell subtypes [28]. Although there is no definite result to prove whether there is a relationship between PKA and lung cancer, previous studies suggest that cAMP signaling pathway may play an important role in the occurrence of lung cancer.

DNA methylation is an important epigenetic mechanism, which is significantly related to lung cancer and plays an important role in the process of carcinogenesis [29, 30]. We identified two lung cancer-specific methylation markers, CFTR and PKIA. The methylation of these genes can be used as potential biomarkers for the diagnosis and prognosis of lung cancer. However, there are still certain limitations, and further biological verification and clinical trials are needed to evaluate the clinical significance of these methylation markers. The results of this study provide relevant ideas for elucidating the development and prognosis of lung cancer and contribute to the early detection and treatment of cancer.

However, the study in the article and its results are only a preliminary study, and the present study has some limitations. First, this study used a sample of LUAD patients from publicly available databases for analysis, and these samples may differ, leading to inconsistent results. Second, although we built and validated prediction models in the TCGA database and the GEO database, further external validation is needed to verify the reliability of these results. In addition, our findings are based on bioinformatics analysis and further clinical trials are needed to validate the prognostic predictive value of these potential biomarkers in real patients. Finally, although our findings suggest that the methylation status of CFTR and PKIA genes is associated with prognosis in LUAD, we need further larger cohort studies to validate these findings. studies to gain insight into the exact mechanisms and mode of action of these genes in LUAD onset and progression.

## Conclusions

Lung cancer is a common malignant tumor with a high mortality rate. Abnormal DNA methylation is closely related to the occurrence and development of cancer. Our study shows that the methylation status of CFTR and PKIA can be used as potential prognostic biomarkers and therapeutic targets in lung cancer, and their prognostic value needs to be further explored to improve the survival prediction of patients.

### Electronic supplementary material

Below is the link to the electronic supplementary material.

Supplementary Material 1

Supplementary Material 2

Supplementary Material 3

Supplementary Material 4

Supplementary Material 5

Supplementary Material 6

Supplementary Material 7

Supplementary Material 8

Supplementary Material 9

## Data Availability

All data and R scripts in this study are available from the corresponding author upon reasonable request. Publicly available datasets were analyzed in this study, these can be found in The Cancer Genome Atlas (https://portal.gdc.cancer.gov/), and Gene Expression Omnibus (https://www.ncbi.nlm.nih.gov/geo/ GSE30219).

## Declarations

**Ethics approval and consent to participate**.

Studies involving human participants were reviewed and approved by the Ethics Committee of Xuzhou Central Hospital. Patients provided written informed consent to participate in this study.

**Consent for publication**.
