# Supplementary figures and images for: Exploring the methylation status of CFTR and PKIA genes as potential biomarkers for lung adenocarcinoma
Source: Orphanet J Rare Dis. 2023 Aug 29;18:246. doi: 10.1186/s13023-023-02807-1 (PMC10466921; doi:10.1186/s13023-023-02807-1)

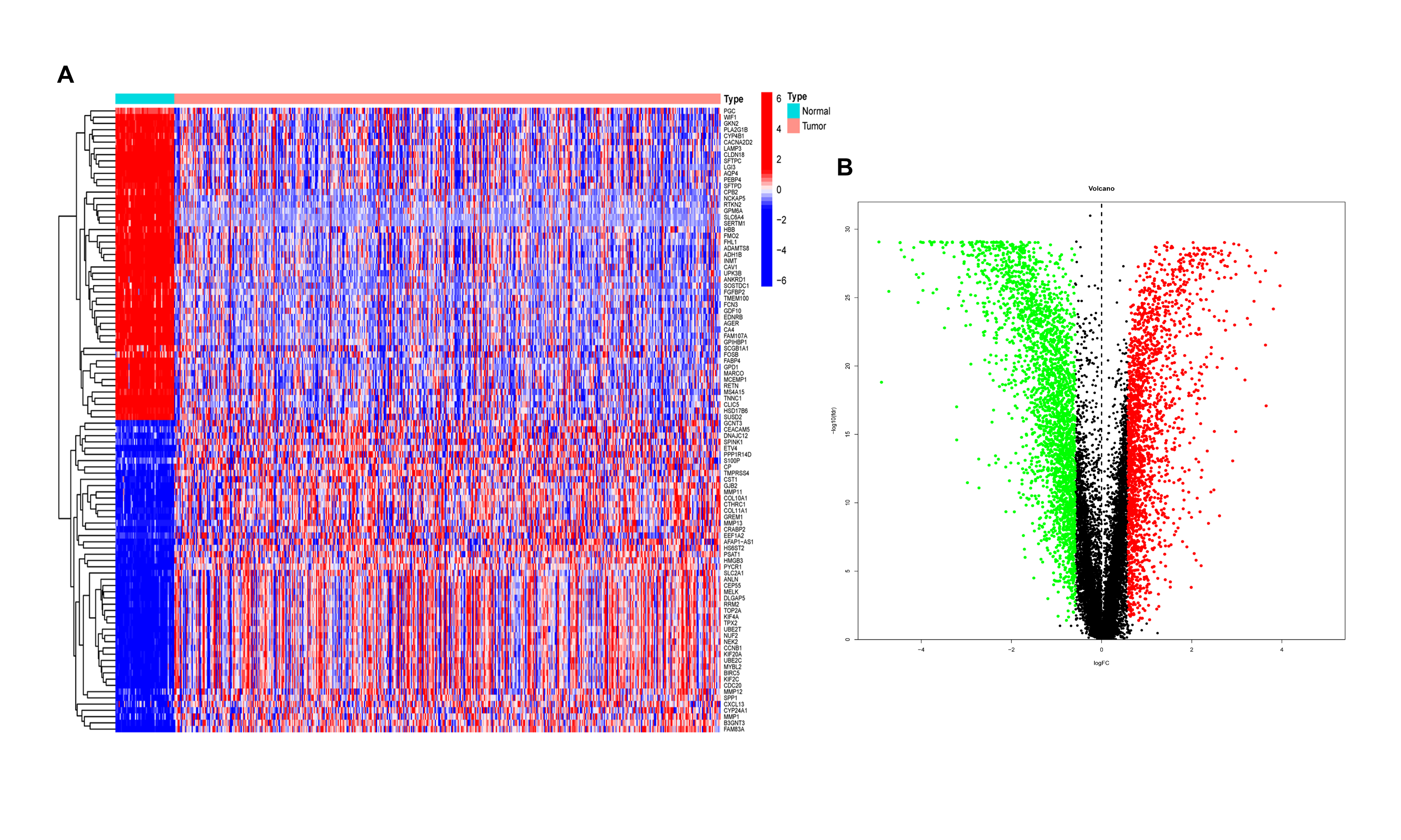

Supplement: Supplementary file 1 — Supplementary Material 1 [file 13023_2023_2807_MOESM1_ESM.tif]

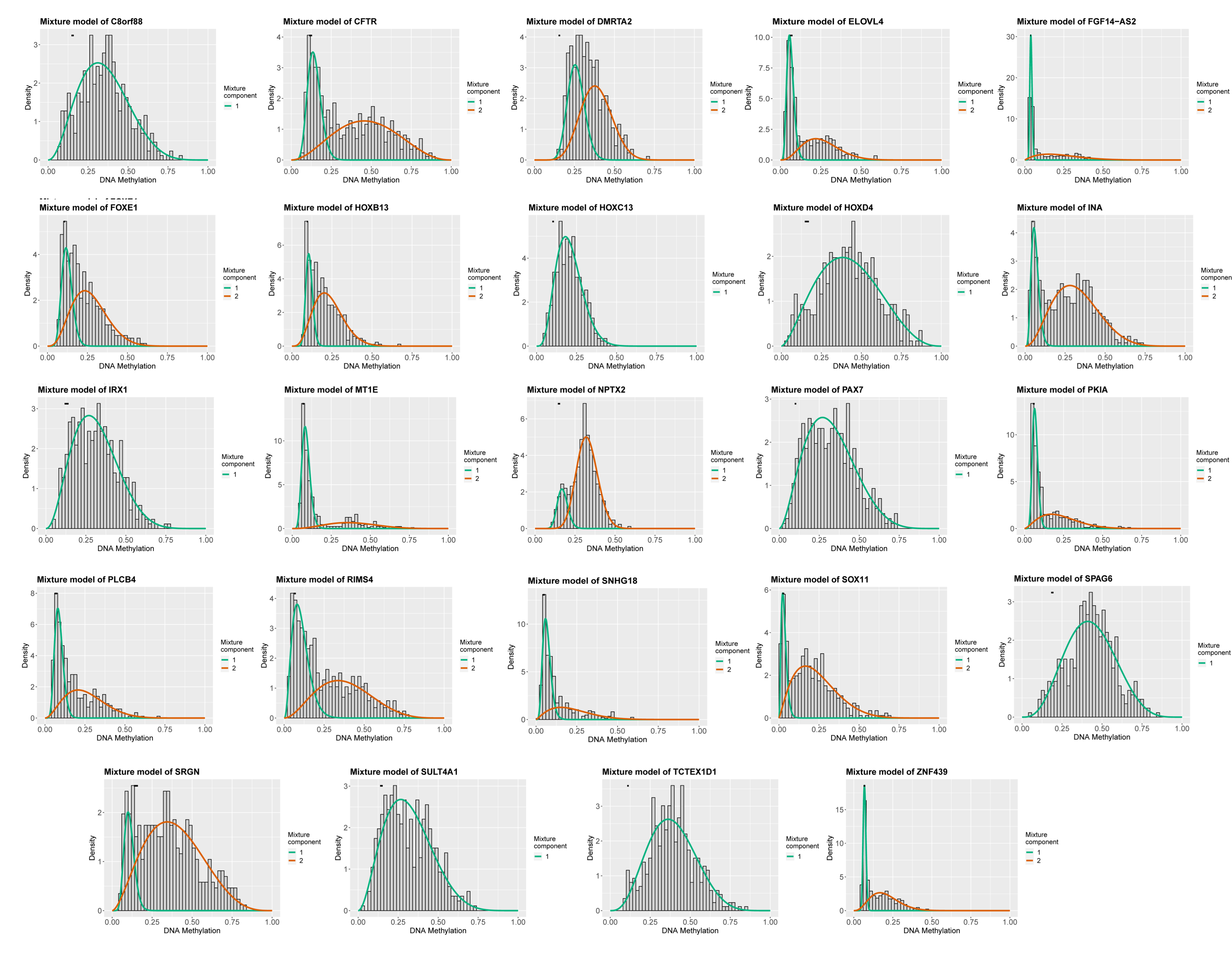

Supplement: Supplementary file 2 — Supplementary Material 2 [file 13023_2023_2807_MOESM2_ESM.tif]

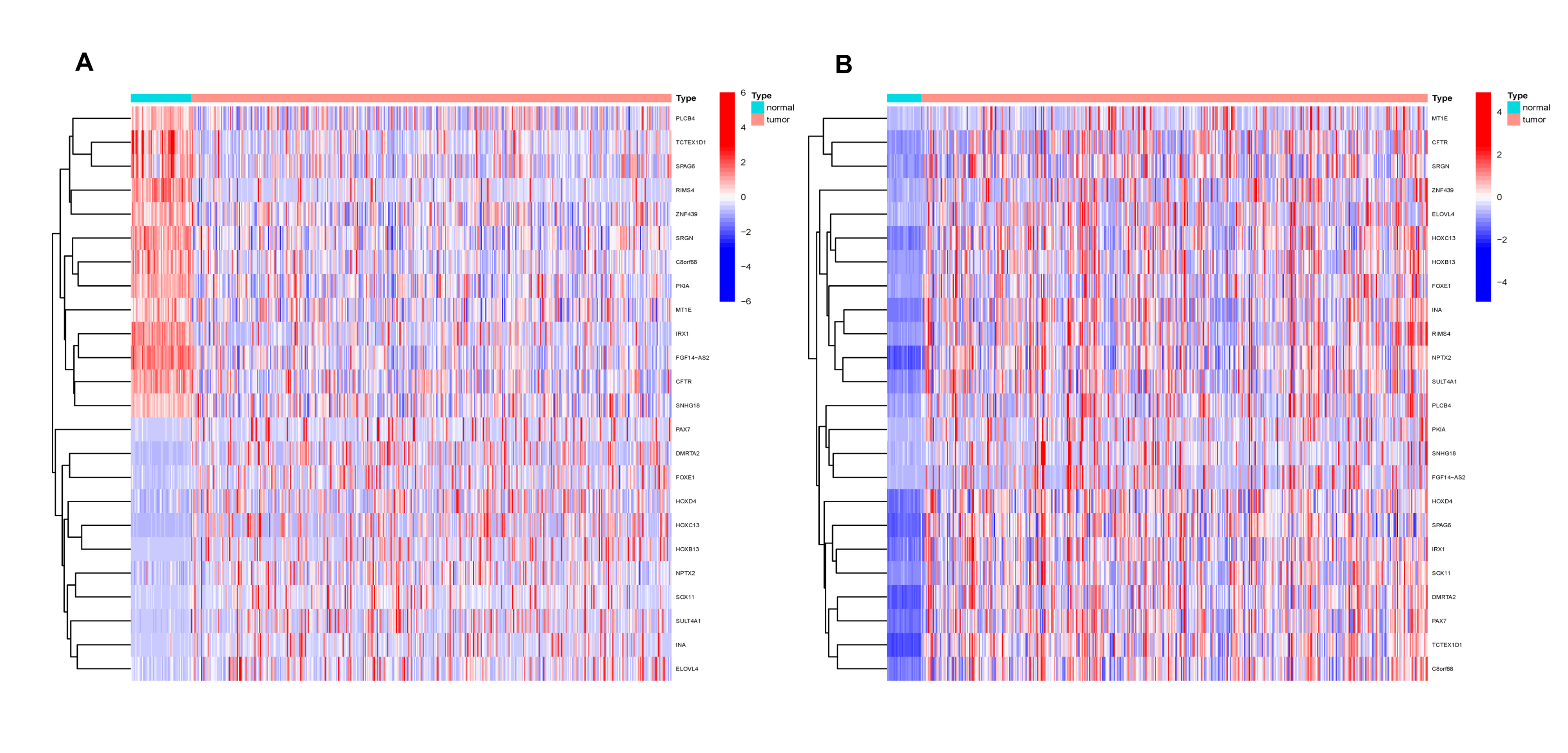

Supplement: Supplementary file 3 — Supplementary Material 3 [file 13023_2023_2807_MOESM3_ESM.tif]

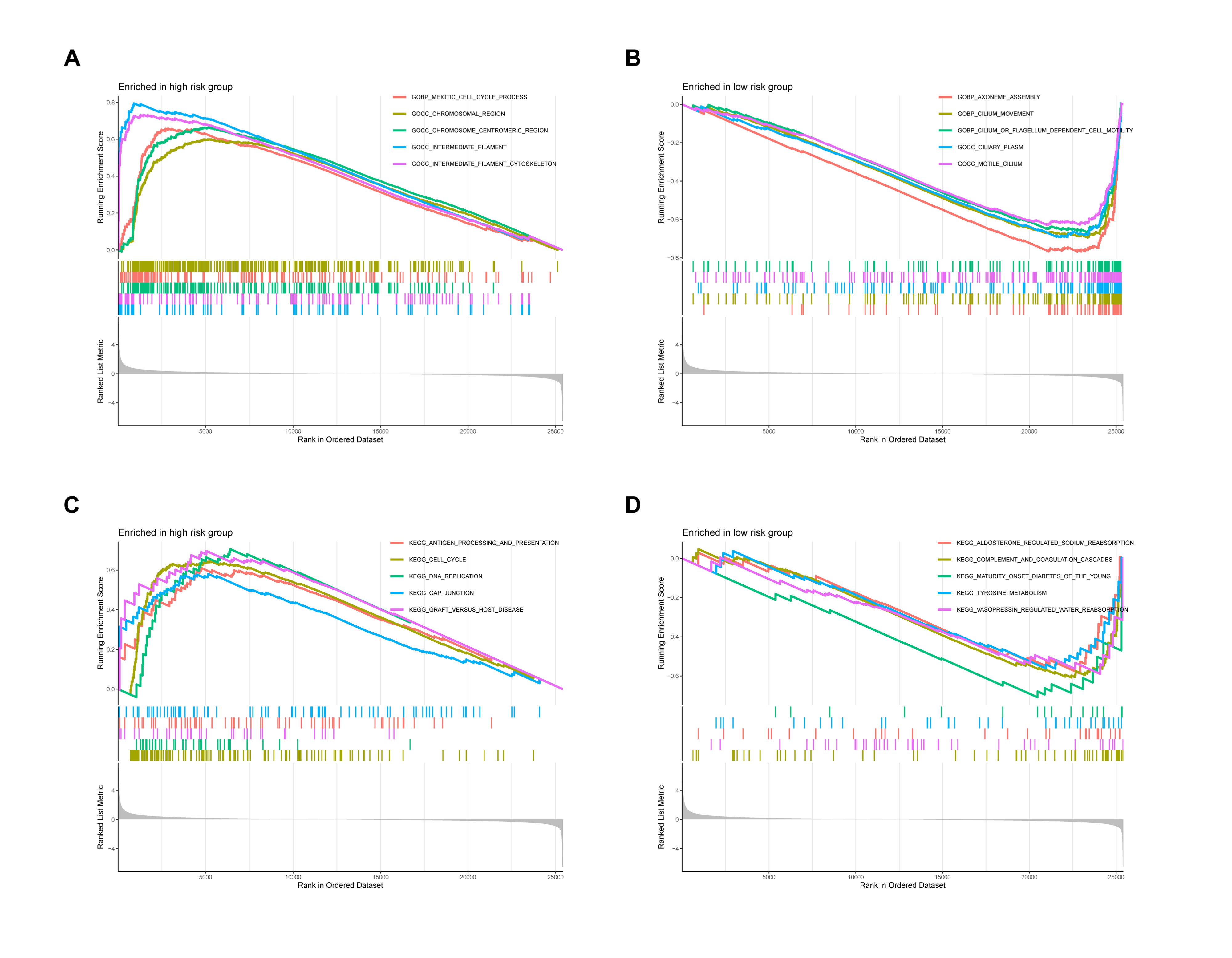

Supplement: Supplementary file 4 — Supplementary Material 4 [file 13023_2023_2807_MOESM4_ESM.tif]
